# Association between root/coronal caries and individual factors in institutionalised elderly using ICDAS severity and activity

**DOI:** 10.1186/s12903-021-01520-4

**Published:** 2021-03-23

**Authors:** Margarita Usuga-Vacca, Dairo Javier Marin-Zuluaga, Jaime Eduardo Castellanos, Stefania Martignon

**Affiliations:** 1 UNICA - Caries Research Unit, Research Department, Universidad El Bosque, Av. Cra. 9 No. 131 A - 02, 110121 Bogotá, Colombia; 2 Research Group in Gerodontology, School of Dentistry, Universidad Nacional de Colombia, Bogotá, Colombia; 3 Grupo de Virología, Vicerrectoría de Investigaciones, Universidad El Bosque, Bogotá, Colombia

**Keywords:** Dental caries, Root caries, Elderly, Health behavior, Partial denture, Health-related quality of life

## Abstract

**Background:**

Caries in the elderly has been associated with dependence, oral-health status and -care practices. This cross-sectional study aimed to investigate the association between root/coronal caries and individual factors among institutionalised elderly people in Bogotá, Colombia, using the International Caries Detection and Assessment System severity and activity criteria (ICDAS).

**Methods:**

A total of 226 institutionalised elderly were clinically examined for root and coronal caries in 40 institutions. Caries risk was assessed with Cariogram, and demographics, oral health knowledge and practices, oral health-related quality of life and denture-use habits using a questionnaire.

**Results:**

Participants (mean age: 80.1 ± 9.3 years; 63.7% female) presented a mean number of 10.8 ± 7.3 teeth and 19.4 ± 18.8 exposed root surfaces. Prevalence of Coronal-ICDAS caries experience (C-ICDAS DF) was of 100% and of 54.4% for C-ICDAS D; mean number of C-ICDAS DFS was 16.76 ± 27.36, with 50.9% of subjects having ≥ one active C-ICDAS DS. Prevalence of Root Caries Index was of 49.1% and of R-ICDAS DF of 46%; mean number of R-ICDAS DFS was 2.03 ± 2.78, with 40.3% of subjects having ≥ one active R-ICDAS DS. Most individuals had a systemic condition (94.2%) and required oral-hygiene assistance (58%). Logistic regression analyses showed significant associations (p < 0.05): for coronal active caries when having over six teeth (OR: 2.7), and for root caries, when having coronal caries (OR: 2.41), being a man (OR: 1.95), and having over 14 teeth (OR: 0.30). Those presenting with > eight exposed root surfaces were 4.04 more likely to have root caries and 2.4 times more likely to have active root caries.

**Conclusion:**

In the institutionalised elderly population in Bogotá significant associations were found, both for the presence as for the activity status of root and coronal caries, with individual clinical factors including coronal caries, exposed root surfaces and number of teeth.

**Supplementary Information:**

The online version contains supplementary material available at 10.1186/s12903-021-01520-4.

## Background

There is a rapid increase in life expectancy throughout the world. The global population aged 60+ years was 962 million in 2017 and is expected to double by 2050, when it is projected to reach nearly 2.1 billion. The proportion of older adults in Colombia is expected to increase from 11.6 to 27.5% during the same period [1]. The more recent National Oral Health Study in Colombia in the elderly (non-institutionalised, 65–79 years) show a high burden of caries experience, with a prevalence of coronal caries experience (DMF) of 96.3%, a mean number of 8.0 ± 0.1 present teeth, a prevalence of cavitated root caries lesions (D) of 31.4%, and a RCI of 0.7 [2].

Geriatric institutions are a more frequently residence option for the elderly and living in these institutions has been described as a factor associated with a high presence of caries [3, 4]. Higher instances of disability, frailty and dependence are common among institutionalised elderly people [5, 6] and are associated with a higher likelihood of having oral health problems, such as dental caries [5, 7]. An increase in the number of retained teeth [7–9] and those with pre-existing root caries lesions, plaque [9–11], coronal caries [12, 13], exposed root surfaces [10–14] and hyposalivation [15] and intake of free sugar [16, 17] have been described as risk factors for root caries [16, 17].

Root Caries Index (RCI) is one of the most frequently used epidemiological indices to measure the root caries experience at the tooth/surface level [18, 19]. This conventional index quantifies in teeth with exposed roots, the cavitated caries lesions (D) and fillings (F).

For coronal caries, the conventional DMF index has been the most widely used [19]. This includes Decayed (D), Missing (M), and Filled (F) tooth/surfaces, considering for D only caries lesions at a cavitated stage [19].

The International Caries Detection and Assessment System (ICDAS) is a detection and assessment system classifying stages of the caries process based on histological extent and activity (when included). Its caries criteria include besides extensive-stage caries lesions (cavitated deep dentine lesions), moderate- and initial-caries lesions (enamel/superficial dentine and non-cavitated lesions). The Conventional DMF index considers as its D component the extensive (and in some studies also the moderate) caries lesions. Thus ICDAS can be used both to compare with other studies using DMF and in addition can separately incorporate the initial caries lesions to have a wider and more current epidemiological caries profile description [20]. ICDAS can also assess the activity/progression status of the caries lesions. Altogether this information allows for a more accurate local and/or national information needs and facilitates a move from an operative/surgical approach to more non-operative/preventive treatment of dental caries [20]. Among the elderly, ICDAS has been used for coronal caries for over a decade [2, 21, 22] and more recently for root caries [13, 22].

The identification of factors associated with root/coronal caries in institutionalised elderly using more sensitive caries criteria and considering the caries lesions’ progression status contributes to guide proposals focused on reducing the caries burden in this population. Thus, the aim of this cross-sectional study was to investigate the association between root/coronal caries and individual factors among institutionalised elderly people in Bogotá, Colombia, using the ICDAS severity and activity criteria.

## Materials and methods

This cross-sectional study counted with IRB (012-2016) and followed the STROBE guidelines.

Based on the number of registered geriatric institutions (n = 152) [23], the estimated universe number of institutionalized elders is of around 1900. The sample size was calculated with the sample calculation software 1.1®, based on the epidemiological data of prevalence of caries experience (DMF coronal caries lesions) of of 96.3% among the elderly obtained from the 2015 National Oral Health Survey [2], anticipating a similar corresponding prevalence and considering a proportion of oversample of 10% for possible drop-out of subjects during acquisition of clinical exam and surveys. Thus, a sample size of 196 elderly adults were to be included in this study.

The inclusion criteria were adults aged 60+ years from institutions who did not present reported terminal illness or severe mental impairment in the medical records, nor edentulism, and who agreed to participate by providing a personal/legal representative signed informed consent forms. The exclusion criterion was mouth opening limitation at the time of the clinical examination.

In November 2016, we received from the District Secretary of Social Integration the list of geriatric institutions in the city of Bogota, indicating 152 registered geriatric institutions [23]. From November 2016 to February 2017, we invited all institutions to participate, via post mail and phone calls. Upon acceptance of institutions, for this study subjects 60+ years old who met the inclusion criteria were personally invited to participate. Subjects from whom we obtained signed consent forms were recruited between March 2017 and May 2018 and data collection was conducted between April 2017 and June 2018.

### Clinical examination

Three examiners who had been previously clinically calibrated for the coronal ICDAS caries visual severity and activity criteria by an ICDAS core examiner (SM) (inter/intra-examiner reproducibility-weighted Kappa values were ≥ 0.7 for severity and ≥ 0.6 for activity) underwent a theoretical and preclinical training and a clinical calibration with natural teeth and elderly patients for root ICDAS caries visual severity and activity criteria by an expert consensus group (MUV, DJMZ, SM), previously discussing and solving difficult cases with the ICDAS core group (inter/intra-examiner reproducibility-weighted Kappa values were ≥ 0.9 for severity and ≥ 0.7 for activity).

After assisted tooth brushing, clinical examinations were conducted in the geriatric institution facilities during the morning hours, assessing the subjects in their beds, wheelchairs, or communal sitting areas, using headlamps, mouth mirrors, tweezers, ball-ended probes, and cotton rolls. The tooth surfaces were dried with cotton rolls for the examination. Data regarding clinical factors, including use of partial/total dentures; the number of teeth; number of exposed root surfaces, thick plaque, hyposalivation, fillings and caries lesions (coronal/root and active/inactive) were collected. The coronal (C) ICDAS_epi_-merged assessment of caries lesions (without air drying) included: C-Sound, C-Initial (C-ICDAS 1–2), C-Moderate (C-ICDAS 3–4) and C-Extensive (C-ICDAS 5–6); activity assessment based on: the severity of caries (non-cavitated vs. cavitated), the colour and opacity of the tooth, the tactile sensation and if located in a plaque stagnation area [15, 24]. The root (R) ICDAS assessment of caries lesions (without air drying) included: R-Sound; R-Initial (R-ICDAS 1); R-Moderate (R-ICDAS 2, ≥ 0.5 to ≤ 2 mm cavitation) and R-Extensive (R-ICDAS 2, > 2 mm cavitation), along with the activity assessment-based on the same criteria as described earlier but for dentine/root, and with the inclusion of the contour of the root [15, 24, 25]. In 10% of the subjects the ICDAS coronal and root caries examinations were repeated.

For the coronal (C) conventional (Co) caries experience, the prevalence/mean number of decayed (D) and filled (F) surfaces (S) (C-Co DFS) was used, where D corresponded to the C-ICDAS-merged-Moderate-Extensive caries lesions; the C-ICDAS-Initial caries lesions were included in the corresponding C-ICDAS caries experience (C-ICDAS DFS).

For the root (R) conventional (Co) caries experience (prevalence/mean number), the RCI was used, which included for its calculation both D (cavitated caries lesions) and F. R-ICDAS-Initial caries lesions were included in the corresponding R-ICDAS caries experience (R-ICDAS DFS).

### Questionnaire on individual factors and caries risk classification

An interview-led questionnaire on individual factors containing 18 items based on validated tools or international guidelines [2, 17, 25–30] was conducted by a trained researcher to assess individual factors (Additional file 1). The 14 general items included in the questionnaire were as follows: demographics (age and gender); condition (presence of hyposalivation); presence of systemic diseases; oral health-related practices (oral hygiene); diet content/frequency habits-assessed by evaluating the daily consumption of free sugars according to the World Health Organization (WHO) guidelines; dental care, related to the last dental visit (reason and time elapsed since); Geriatric/General Oral Health Assessment Index (GOHAI) (to assess the physical functions such as oral hygiene assistance, pain or discomfort, painful sites and use of medication and psychosocial functions such as worry/concern about teeth/gums/dentures and perceived need for dental care). Four additional items were included for those wearing denture, about their oral health-related practices (denture wear time, denture daily cleaning and denture removing before bedtime).

The individual caries risk was calculated using the Cariogram software [27, 29] using information from both the questionnaire and the results of the clinical assessment. Other factors from the CariesCare International consensus [15], such as ‘Regular preventive-oriented dental care’ and ‘Symptomatic-driven dental attendance’, were assessed using the Cariogram clinical judgement component, where possible. The subjects were classified into high-risk (moderate and high) and low-risk groups.

### Statistical analyses

The categorical variables are expressed in terms of the distribution of absolute and relative frequencies and the continuous variables as measures of central tendency (mean and standard deviations). The subjects’ cut off points for both ICDAS coronal and ICDAS root caries were: those without presence and those with presence of ICDAS caries lesions, and those without presence and those with presence of ICDAS active caries lesions. The associations between the individual factors investigated and the outcome measures: presence of ≥ 1 root caries lesion; ≥ 1 active root caries lesion; ≥ 1 coronal caries lesion, and ≥ 1 active coronal caries lesion, were analyzed using the chi-square test and Fisher's exact test.

In the absence of previously established intrinsic categories the variables "number of teeth in the mouth" and "number of exposed root surfaces" were analyzed using the approach to data analysis by tertiles exposure [31, 32].

The first step of the analyses was crude logistic regression analysis. Subsequently, non-conditional multivariate logistic regression models were used to estimate the odds ratio and 95% confidence intervals (CI) between the individual variables investigated and the presence of coronal ICDAS/root ICDAS caries lesions, and active coronal ICDAS/active root ICDAS caries lesions, respectively. Furthermore, the relative effect of each individual factor on caries risk was adjusted for age, sex, systemic disease, daily sugar-free frequency intake, thick dental plaque, dental floss use, fluoridated toothpaste use, dental-pain/discomfort medication/food-type change during the last 3 months.

Final multivariate models were created through stepwise elimination of variables of interest from bivariate analysis. Main independent variables and the covariates were incorporated in the multivariate logistic regression model, if they had a significant (p < 0.05) or marginal association (p < 0.20) with the outcome measures, while variables considered in the literature or contextually as clinically and biologically relevant were retained. These included age to compare at a national level with corresponding ages [2]; hyposalivation due to its relevance as a caries risk factor [15], as well as daily sugar intake [15, 17], and recent concerns due to dental problems as a quality of life indicator [30]. P values of less than 0.05 for associations were considered to indicate statistical significance. SPSS Statistics V.23 (IBM SPSS Statistics) and Stata® 12 (StataCorp LLC) were used for statistical analyses.

## Results

Out of 152 geriatric institutions in Bogotá, 52 were not reachable after sending post mail and at least three phone calls, and 60 did not agree to participate in the study. In total, 40 geriatric institutions located in five of the 20 municipalities in Bogotá accepted the invitation (26.3%). From a total of 468 elderly geriatric-institution residents, 242 were not included (51.7%): 15 did not agree to participate; 37 were not within the age range; 15 were terminally ill, and 174 were edentulous. The remaining 226 agreed to participate in the study providing signed consent forms and none were excluded at the clinical examination, for a total examined sample of n = 226. The mean age of the subjects was 80.1 ± 9.3 years; 63.7% were women.

The subjects presented with a mean number of 10.8 ± 7.3 teeth (range, 1–28) and a population distribution of 1–6 teeth (35.4%), 7–14 teeth (30.1%) and 15–28 teeth (96.3%). The mean number of exposed root surfaces was 19.4 ± 18.8 (range, 0–96) with a population distribution as follows: 0–8 exposed surfaces (36.7%), 9–21 exposed surfaces (29.3%) and 22–96 exposed surfaces (34.9%). The majority (n = 222; 98.7%) had partial tooth loss (67.7% in both jaws and 31% only in the lower jaw); 30.5% (n = 69) of the subjects wore removable partial dentures (7.1% in both jaws, 16.3% only in the lower jaw and 7.1% only in the upper jaw). A total of 80 subjects (35.4%) were edentulous only in one jaw (28.8% upper jaw and 6.6% lower jaw), whereas 25.2% of them wore complete dentures (upper: 22.1% and lower: 3.1%).

The prevalence of C-Co DF was 83.6% and that of C-Co D was 46.5%, which increased to 54.4% with the inclusion of Initial C-ICDAS D. The mean number of C-ICDAS-DFS was 16.76 ± 27.36 (Initial DS, 0.4 ± 0.89; Moderate-Extensive DS, 4.6 ± 10.5; and FS, 11.8 ± 16.4). Around half of the subjects (50.9%) showed at least one coronal ICDAS active caries lesion with a mean number of 4.7 ± 9.6 involved surfaces.

Concerning root caries, the prevalence of RCI root caries was 49.1%, with a mean number of 1.09 ± 2.24 involved surfaces. The prevalence of R-ICDAS DF was 46%, and the mean number of R-ICDAS DFS was 2.03 ± 2.78 (Initial DS: 0.62 ± 1.21; Moderate-Extensive DS: 0.80 ± 1.67; and FS: 0.58 ± 1.23). Over a third of the subjects (40.3%) showed at least one active root ICDAS caries lesion and a mean number of 1.25 ± 2.44 involved surfaces.

The repetition of clinical coronal and root ICDAS caries examinations reported perfect intra-examiners’ agreement of 78.5% for coronal ICDAS and of 91.0% for root ICDAS.

The questionnaire showed that most of the participants had a systemic condition (94.2%), had last visited the dentist over a year ago (84.1%) and reported daily free sugar consumption above the recommended level (77.4%). Furthermore, 58% of the subjects required assistance to perform oral hygiene. Among those wearing denture (n = 69), 79.7% used it for < 2 years, 75.4% cleaned it at least once a day, with dentifrice/mouthrinse (97.1%), and 50.7% did not remove it before bedtime. Over a third of the population perceived the need for dental care (35.4%) and reported emergency as the reason for the last dental visit (38.1%). A small number reported the presence of painful sites in the mouth (5.3%) or were diagnosed with hyposalivation (3.1%).

Table 1 shows with the bivariate model statistical associations between the number of teeth in the mouth and presence of coronal ICDAS/coronal ICDAS active caries lesions, between exposed root surfaces and presence of root ICDAS/root ICDAS active caries lesions and between 1/day-denture cleaning and root ICDAS caries (*p* < 0.05).

**Table 1:** Association of individual factors and root/coronal ICDAS active/inactive caries lesions

	Presence of ≥ 1 root caries lesions			Presence of ≥ 1 active root caries lesions			Presence of ≥ 1 coronal caries lesions			Presence of ≥ 1 active coronal caries lesions		
	No (n = 122)	Yes (n = 104)	p value	No (n = 135)	Yes (n = 91)	p value	No (n = 103)	Yes (n = 123)	p value	No (n = 111)	Yes (n = 115)	p value
	n (%)	n (%)		n (%)	n (%)		n (%)	n (%)		n (%)	n (%)	
Sex*												
Female	84 (68.9)	60 (57.7)	0.082	90 (66.7)	54 (37.5)	0.261	65 (63.1)	79 (64.2)	0.861	71 (64.0)	73 (63.5)	0.939
Male	38 (31.1)	44 (42.3)		45 (54.9)	37 (45.1)		38 (36.9)	44 (35.8)		40 (36.0)	42 (36.5)	
Age (years)*												
< 65	11 (9.0)	11 (10.7)	0.693	12 (54.5)	10 (45.4)	0.601	8 (7.8)	14 (11.4)	0.361	10 (9.0)	12 (10.4)	0.718
≥ 65	111 (91.1)	93 (89.4)		123 (60.3)	81 (39.7)		95 (92.2)	109 (88.6)		101 (91.0)	103 (89.6)	
Reported hyposalivation in the medical health record**												
No	119 (97.5)	100 (96.2)	0.549	131 (59.8)	88 (40.2)	1.000	102 (99.0)	117 (95.1)	0.091	107 (96.4)	112 (97.4)	0.666
Yes	3 (2.5)	4 (3.8)		4 (57.1)	3 (42.9)		1 (1.0)	6 (4.9)		4 (3.6)	3 (2.6)	
Caries risk classification**												
Low	49 (40.2)	50 (48.1)	0.232	4 (80.0)	1 (20.0)	0.651	47 (45.6)	52 (42.3)	0.613	50 (45.0)	49 (42.6)	0.712
High	73 (59.8)	54 (51.9)		131 (59.3)	90 (40.7)		56 (54.4)	71 (57.7)		61 (55.0)	66 (57.4)	
Use of partial dentures*^△^												
No	88 (72.1)	69 (66.3)	0.347	96 (71.1)	61 (67.0)	0.514	64 (62.1)	93 (75.6)	0.028	66 (59.5)	91 (79.1)	0.001
Yes	34 (27.9)	35 (33.7)		39 (28.9)	30 (33.0)		67 (65.0)	79 (64.2)		74 (66.7)	72 (62.6)	
Number of toothbrushings per day with ≥ 1000 ppm F^−^ toothpaste*												
≥ 2	72 (59.0)	51 (49.0)	0.133	79 (58.5)	45 (49.5)	0.179	59 (57.3)	64 (52.0)	0.430	62 (55.9)	61 (53.0)	0.671
< 2	50 (41.0)	53 (51.0)		56 (41.5)	46 (50.5)		44 (42.7)	59 (48.0)		49 (44.1)	54 (47.0)	
Minimum of one daily cleaning of the partial denture**^△^												
No	4 (5.8)	11 (15.9)	0.047	7 (10.1)	8 (11.6)	0.384	10 (14.5)	5 (7.2)	0.370	11 (15.9)	4 (5.8)	0.455
Yes	30 (43.5)	24 (34.8)		32 (46.4)	22 (31.9)		29 (42.0)	25 (36.2)		34 (49.3)	20 (29.0)	
Need of assistance to perform oral hygiene*												
No	54 (44.3)	41 (39.4)	0.463	58 (61.0)	37 (38.9)	0.731	48 (46.6)	47 (38.2)	0.203	52 (46.8)	43 (37.4)	0.150
Yes	68 (55.7)	63 (60.6)		77 (58.8)	54 (41.2)		55 (53.4)	76 (61.8)		59 (53.2)	72 (62.6)	
Daily intake of free sugars exceeding the WHO recommendations*												
No	29 (23.8)	22 (21.2)	0.639	30 (58.8)	21 (41.2)	0.880	25 (24.3)	26 (21.1)	0.575	27 (24.3)	24 (20.9)	0.575
Yes	93 (76.2)	82 (78.8)		105 (60.0)	70 (40.0)		78 (75.7)	97 (78.9)		84 (75.7)	91 (79.1)	
Presence of painful sites in the mouth*												
No	118 (96.7)	96 (92.3)	0.140	130 (60.7)	84 (39.2)	0.232	99 (96.1)	115 (93.5)	0.382	107 (96.4)	107 (93.0)	0.382
Yes	4 (3.3)	8 (7.7)		5 (41.7)	7 (58.3)		4 (3.9)	8 (6.5)		4 (3.6)	8 (7.0)	
Number of teeth*												
≤ 6	43 (35.2)	37 (35.6)	0.077	50 (37.0)	30 (32.9)	0.246	46 (44.7)	34 (27.6)	0.028	50 (45.0)	30 (26.1)	0.012
7–14	30 (24.6)	38 (36.5)		35 (25.9)	33 (36.3)		27 (26.2)	41 (33.3)		28 (25.2)	40 (34.8)	
> 14	49 (40.2)	29 (27.9)		50 (37.0)	28 (30.8)		30 (29.1)	48 (39.0)		33 (29.7)	45 (39.1)	
Number of exposed root surfaces												
≤ 8	59 (48.4)	24 (23.1)	0.000	61 (45.2)	22 (24.2)	0.004	44 (42.7)	39 (31.7)	0.227	46 (41.4)	37 (32.2)	0.286
9–21	25 (20.5)	39 (37.5)		31 (22.9)	33 (36.3)		27 (26.2)	37 (30.1)		31 (27.9)	33 (28.7)	
≥ 22	38 (31.1)	41 (39.4)		43 (31.9)	36 (39.6)		32 (31.1)	47 (38.2)		34 (30.6)	45 (39.1)	
*During the last three months have you …*												
been concerned about dental problems?**												
No	114 (93.4)	98 (94.2)	0.806	127 (59.6)	86 (40.4)	0.891	96 (93.2)	116 (94.3)	0.731	103 (92.8)	109 (94.8)	0.731
Yes	8 (6.6)	6 (5.8)		8 (61.5)	5 (38.5)		7 (6.8)	7 (5.7)		8 (7.2)	6 (5.2)	
used medication due to dental pain or discomfort?**												
No	121 (99.2)	100 (96.2)	0.123	134 (60.6)	87 (39.4)	0.160	102 (99.0)	119 (96.7)	0.246	110 (99.1)	111 (96.5)	0.246
Yes	1 (0.8)	4 (3.8)		1 (20.0)	4 (80.0)		1 (1.0)	4 (3.3)		1 (0.9)	4 (3.5)	
changed the type of food due to dental pain or discomfort?**												
No	115 (94.3)	94 (90.4)	0.271	127 (94.1)	82 (90.1)	0.268	98 (95.1)	111 (90.2)	0.164	104 (93.7)	105 (91.3)	0.164
Yes	7 (5.7)	10 (9.6)		8 (5.9)	9 (9.9)		5 (4.9)	12 (9.8)		7 (6.3)	10 (8.7)	

*χ^2^ test; **Fisher test. ^△^Data established based on subjects that wear partial denture (n = 69)

Table 2 highlights with the logistic regression model significant associations between having over 6 teeth and coronal ICDAS active caries lesions (OR 2.84; *p* < 0.05) and between having over 8 exposed root surfaces (OR 3.01; *p* < 0.05) and root ICDAS active caries lesions.

**Table 2:** Associations between coronal ICDAS active caries lesions, root ICDAS active caries lesions and individual factors

	Presence of ≥ 1 active coronal caries lesions		Presence of ≥ 1 active root caries lesions	
	Crude model	Adjusted model	Crude model	Adjusted model
	OR [95% CI]	OR [95% CI]	OR [95% CI]	OR [95% CI]
Sex				
Female	1 [Reference]	1 [Reference]	1 [Reference]	1 [Reference]
Male	1.02 [0.57–1.82]	0.84 [0.46–1.54]	1.37 [0.76–2.46]	1.33 [0.72–2.46]
Age (years)				
< 65	1 [Reference]	1 [Reference]	1 [Reference]	1 [Reference]
≥ 65	0.84 [0.31–2.25]	1.00 [0.37–2.73]	0.79 [0.29–2.14]	0.82 [0.30–2.26]
Reported hyposalivation in the medical health record				
No	1 [Reference]	1 [Reference]	1 [Reference]	1 [Reference]
Yes	0.71 [0.10–4.34]	0.34 [0.05–2.16]	1.11 [0.15–6.77]	0.50 [0.04–3.38]
Caries risk classification				
Low	1 [Reference]	1 [Reference]	1 [Reference]	1 [Reference]
High	1.10 [0.63–1.93]	1.33 [0.63–2.00]	0.73 [0.41–1.30]	0.89 [0.49–1.59]
Number of teeth in the mouth				
≤ 6	1 [Reference]	1 [Reference]	1 [Reference]	1 [Reference]
7–14	2.38 [1.22–4.61]	2.84 [1.31–6.15]	1.57 [0.81–3.02]	1.25 [0.56–2.77]
> 14	2.27 [1.20–4.30]	2.78 [1.27–6.09]	0.93 [0.48–1.78]	0.70 [0.31–1.59]
Number of toothbrushings per day with ≥ 1000 ppm F^−^ toothpaste				
< 2	1 [Reference]	1 [Reference]	1 [Reference]	1 [Reference]
≥ 2	1.16 [0.66–2.03]	1.44 [0.75–2.77]	1.50 [0.85–2.66]	1.42 [0.73–2.76]
Daily intake of free sugars exceeding WHO recommendations				
No	1 [Reference]	1 [Reference]	1 [Reference]	1 [Reference]
Yes	1.21 [0.62–2.39]	1.36 [0.64–2.90]	0.95 [0.48–1.90]	0.75 [0.35–1.59]
Painful sites in the mouth				
No	1 [Reference]	1 [Reference]	1 [Reference]	1 [Reference]
Yes	2.00 [0.51–9.32]	1.92 [0.45–8.15]	2.16 [0.56–8.92]	2.18 [0.54–8.75]
Number of exposed root surfaces				
≤ 8	1 [Reference]	1 [Reference]	1 [Reference]	1 [Reference]
9–21	1.32 [0.68–2.54]	1.08 [0.54–2.48]	2.95 [1.47–5.89]	3.01 [1.41–6.40]
> 21	1.64 [0.88–3.06]	1.16 [0.54–2.48]	2.32 [1.20–4.48]	2.42 [1.07–5.46]

1. The model was also adjusted for other variables without statistical significance and higher p values: Systemic disease, Daily diet frequency intake, Thick dental plaque, Dental floss use, F^−^ toothpaste use, Dental-pain/discomfort medication/food-type change during last 3 months

2. The variable “Need of assistance to perform oral hygiene” was omitted due to collinearity

In Table 3, the results of the logistic regression model and the associations between the presence of root ICDAS caries lesions/root ICDAS caries active lesions and the individual variables, including the presence of coronal ICDAS caries lesions and coronal ICDAS active caries lesions.

**Table 3:** Associations between root ICDAS caries lesions, root ICDAS active caries lesions and individual factors

	Presence of ≥ 1 root caries lesions		Presence of ≥ 1 active root caries lesions	
	Crude model	Adjusted model	Crude model	Adjusted model
	OR [95%CI]	OR [95%CI]	OR [95%CI]	OR [95%CI]
Sex*				
Female	1 [Reference]	1 [Reference]	1 [Reference]	1 [Reference]
Male	1.62 [0.90–2.90]	1.95 [1.05–3.61]	1.37 [0.76–2.46]	1.44 [0.80–2.60]
Age* (years)				
< 65	1 [Reference]	1 [Reference]	1 [Reference]	1 [Reference]
≥ 65	0.83 [0.31–2.23]	0.83 [0.31–2.22]	0.70 [0.29–2.14]	0.81 [0.31–2.11]
Caries risk classification				
Low	1 [Reference]	1 [Reference]	1 [Reference]	1 [Reference]
High	0.72 [0.41–1.27]	0.81 [0.45–1.45]	0.73 [0.41–1.30]	0.82 [0.47–1.44]
Presence of thick plaque				
No	1 [Reference]	1 [Reference]	1 [Reference]	1 [Reference]
Si	1.22 [0.69–2.15]	1.43 [0.77–2.64]	1.09 [0.61–1.95]	1.20 [0.66–2.1]
Partial denture wear**^△^				
No	1 [Reference]	1 [Reference]	1 [Reference]	1 [Reference]
Yes	1.31 [0.71–2.40]	1.29 [0.67–2.48]	1.21 [0.65–2.32]	1.26 [0.66–2.40]
Presence of coronal caries lesions				
No	1 [Reference]	1 [Reference]	NI	NI
Yes	2.13 [1.20–3.78]	2.41 [1.33–4.39]	NI	NI
Presence of active coronal caries lesions				
No	NI	NI	1 [Reference]	1 [Reference]
Yes	NI	NI	1.64 [0.92–2.91]	1.70 [0.95–3.04]
Number of teeth in the mouth				
≤ 6	1 [Reference]	1 [Reference]	1 [Reference]	1 [Reference]
7–14	1.47 [0.76–2.81]	0.82 [0.37–1.80]	1.57 [0.81–3.00]	1.04 [0.49–2.24]
> 14	0.68 [0.36–1.29]	0.30 [0.13–0.70]	0.92 [0.48–1.78]	0.58 [0.26–1.28]
Exposed root surfaces				
≤ 8	1 [Reference]	1 [Reference]	1 [Reference]	1 [Reference]
9–21	3.83 [1.92–7.65]	4.04 [1.88–8.66]	2.95 [1.47–5.89]	2.82 [1.36–5.85]
≥ 22	2.65 [1.38–5.07]	3.51 [1.56–7.90]	2.32 [1.20–4.48]	2.39 [1.10–5.21]

* χ^2^ test; ** Fisher test. ^△^Data established based on subjects that wear partial denture (n = 69). *NI* not included

Those who had coronal caries lesions and were men, were correspondingly 2.41, and 1.95 times more likely to have root ICDAS caries lesions. Furthermore, those who had over 21 exposed root surfaces were 3.51 times more likely to have root ICDAS caries lesions and 2.39 times more likely to have root ICDAS active caries lesions. Conversely, those who had over 14 teeth were 70% less likely to have root ICDAS caries lesions.

## Discussion

In general, most caries occurs in adulthood [33] and the institutionalised population has conditions that make them particularly vulnerable to caries [5, 7].

This cross-sectional study was carried out in 40 geriatric institutions in the city of Bogotá, Colombia.

The institutionalised elderly population presented with a high coronal and root caries experience and high frequency of coronal/root ICDAS active caries lesions, which were associated with individual factors. The reported association between coronal caries and root caries [12, 13, 34] was confirmed in this study with the ICDAS criteria. A stronger correspondent association was found by Hayes et al*.* [13] (OR 4.50) (Root Decay Filled Surfaces index) and a weaker one (OR 1.03) by Nicolau et al*.* [8] (DMFS index). These differences might be partly explained by the discrepancies in the indices used.

The significant association between the presence of > 6 teeth and coronal ICDAS active caries lesions as much as the presence of > 8 root surfaces and root caries could be explained by the retention of a considerable number of tooth surfaces that increases the likelihood of caries lesions development in coronal and root surfaces, similar to what was reported by different authors [7, 10–13, 35]. This highlights the need for better oral hygiene and biofilm control with fluoridated toothpaste use of at least 1000 ppm F^−^ concentration, as well as sugars control within the elderly [14, 15, 17]. Furthermore, the presence of over 14 remainder teeth was found to be a protective factor for root caries (Table 3). Similar to our findings, Fure and Zickert (1990) reported a negative correlation between root caries and number of remaining teeth [36]. This might be related to the presence of teeth without root exposure [10–13, 35] or, as stated by Fure and Zickert (1990), the presence of teeth with available surfaces is not a sufficient factor for the development of caries [36]. Nevertheless, this finding is contrary to that reported by Ritter et al. [9], who reported a higher association between number of teeth and high incidence of root caries.

In the current study, age was not a factor that could be significantly associated with the presence of caries unlike that reported by Ritter et al. for root caries [9].

The ICDAS caries criteria have scarcely been used for root caries epidemiological studies [22]. The advantages of using these criteria versus the WHO criteria, rely on the inclusion of initial/non-cavitated caries lesions and their activity status, in agreement with the current understanding of the caries process and translating it into a more comprehensive and accurate description of the individuals' and population's caries status [15, 25, 37]. Reporting the caries prevalence using the ICDAS criteria provides a more accurate picture of the caries situation in a population in comparison to using the DMFT/S index or the RCI index.

In the study conducted by Christensen et al. [38], they assessed active root caries as lesions located on root surfaces with a soft or leathery texture based on visual–tactile examination [15, 24]. In the current study, we assessed the severity (initial, moderate, and extensive) and activity (active or inactive) of the lesions, in addition to the *visual–tactile examinations*. This discrimination gave us the possibility to compare the presence of at least one root caries lesion (active/inactive) and at least one active caries lesion with the variables in this study. A 10 times higher prevalence of active caries lesions (40.3%) was observed in the current study when compared with that from Denmark (3.7%) [38], which might reflect a less comprehensive national health system because the Colombian study included people 60 + only a dental appointment every two years including professional prophylaxis, calculus removal and oral hygiene instructions [39]. Another explanation for the difference in the prevalence between the two studies is the fact that we examined only institutionalised elderly people who presented with higher caries root experience [5, 6, 40]. Moreover, we used the root ICDAS caries criteria including different severity scores and active/inactive caries lesions, whereas the study by Christensen et al. [38] included only one type of root caries lesions, namely, the active root caries, defined as localised caries lesions in root surfaces with a soft/leathery texture [25, 38].

On average in our study we a higher mean number of remaining teeth (10.8) in comparison to that of 8.0 in the most recent Colombian National Oral Health Study [2]. However, in neither of the two studies the minimum number of 10 teeth per arch to ensure chewing efficiency was achieved [41].

The variable ‘Not cleaning the denture at least once a day’ was the only one that showed any association with root active caries lesions, possibly indicating better self-care. Unlike other studies, we did not find any association between caries and the dependence to deliver oral hygiene [5, 6, 42]. Furthermore, similar to the study by Christensen et al. [38], no association between caries and high sugar intake was observed, despite the thorough assessment of daily intake of free sugars was performed based on the WHO recommendations in the present study [16, 17].

It is important to note that the results of this study have to be interpreted in the light of institutionalised elderly population. The selected sample represents a higher-than-usual social-economic level within the institutionalised elderly population according to their location within the city in 5 out of 20 municipalities [23]. About one-third of the elderly population from Bogotá live in these five municipalities (33.2%) [23]. This means that the results cannot be considered fully representative of the city of Bogotá. The institutions' rejection to participate in surveys or research projects has been related to low standards of care that exist in many geriatric institutions in Colombia [23]. This is a characteristic of an under-developed country, which helps such institutions to avoid any kind of audit. This issue might also explain the fact that most of the institutions that accepted to participate in this project were located in high social class/income neighbourhoods and that our sample had more teeth than the mean numbers reported in the Colombian population. Nonetheless, the oral health data are still considered to be of value because this is the first study in Colombia to report epidemiological data among institutionalised elderly people. Additionally, severity and activity assessments of both root and coronal caries have been included in this study [15, 37], and detailed information about individual factors have been provided [38]. In addition, there should not be an influence of the lack of generalizability in the validity of the biological and social associations between coronal/root caries and individual variables.

Although logistic regressions can overestimate the effect size of statistical associations under a cross-sectional design and are considered a limitation of this study, we chose this type of statistical analysis following same design conducted by Christensen et al. [38] who also assessed risk factors and root caries. In addition, in this study the cut off point for classifying individuals with and without coronal/root ICDAS caries lesions and with and without coronal/root ICDAS active caries lesions was very rigorous as examiners were highly trained. The other aspect we considered was the fact that the dichotomization of a continuous or discrete predictor can be used in statistical models to simplify the interpretation [43].

Finally, the classification of elderly into with and without caries groups could guide oral care activities. The coordinators and caregivers of geriatric institutions, with the support of health entities, could apply measures that help reduce the prevalence and development of new caries lesions in the affected group, while keeping the "no cavities" group healthy. Taking into account the challenges that the rapid increase in life expectancy with higher number of retained teeth pose, as well as the raise of other health and quality of life aspects, there is an increasing need of education within the dental profession in the comprehensive understanding of the pathology within other oral and general health aspects, as well as current individual- and tooth-surface level caries care guidelines [7, 11, 15, 20, 25, 33, 37, 44].

## Conclusions

In the institutionalised elderly population in Bogotá significant associations were found, both for the presence as for the activity status of root and coronal caries, with individual clinical factors including coronal caries, exposed root surfaces and number of teeth.

## Supplementary Information

**Additional file 1:** Questionnaire on individual factors.

## Data Availability

The datasets used and/or analysed during the current study are available from the corresponding author on reasonable request.

## Abbreviations

R: Root
RCI: Root Caries Index
D: Decayed
F: Filled
DFT: Decayed, missing, filled teeth
DFS: Decayed, missing, filled surfaces
ICDAS: International Caries Detection and Assessment System
C: Coronal
Co: Coronal conventional
IRB: Institutional Review Board
WHO: World Health Organization
GOHAI: Geriatric/General Oral Health Assessment Index
DANE: National Administrative Department of Statistics (for its initials in Spanish, Colombia
