# Supplementary material for: Association between root/coronal caries and individual factors in institutionalised elderly using ICDAS severity and activity
Source: BMC Oral Health. 2021 Mar 23;21:146. doi: 10.1186/s12903-021-01520-4 (PMC7986499; doi:10.1186/s12903-021-01520-4)
Supplement: Supplementary file 1 — Additional file 1: Questionnaire on individual factors. [file 12903_2021_1520_MOESM1_ESM.docx]

**Association between root/coronal caries and individual factors in institutionalised elderly using ICDAS severity and activity**

Margarita Usuga-Vacca^a^, Dairo Javier Marin-Zuluaga^b^, Jaime Eduardo Castellanos^c^, Stefania Martignon^a^

^a^ UNICA - Caries Research Unit, Research Department, Universidad El Bosque, Bogotá, Colombia

^b^ Research Group in Gerodontology, School of Dentistry, Universidad Nacional de Colombia, Bogotá, Colombia

^c^ Grupo de Virología, Vicerrectoría de Investigaciones, Universidad El Bosque. Bogotá, Colombia

Short Title: ICDAS root/coronal caries and individual factors in institutionalised elderly.

**Corresponding Author:**

Stefania Martignon

UNICA - Caries Research Unit

Research Department, Universidad El Bosque

Av. Cra. 9 No. 131 A – 02

Bogotá 110121, Colombia

Tel: + 57 1 6489000 Ext. 1279

E-mail: [martignonstefania@unbosque.edu.co](mailto:martignonstefania@unbosque.edu.co)

**Additional file 1.** Questionnaire on individual factors.

| **Nr.** | **Questions** | **Answer options** |
| --- | --- | --- |
| 1 | What is your gender? | Female |
|  |  | Male |
| 2 | How old are you? | < 65 |
|  |  | ≥ 65 |
| 3 | Do you have a systemic disease (confirmed within the medical record)? | Yes |
|  |  | No |
| 4 | Do you suffer from hyposalivation (confirmed within the medical record)? | Yes |
|  |  | No |
| 5 | When was the last time you visited to the dentist? | During the last year |
|  |  | Over a year ago |
| 6 | Does your free-sugar daily consumption exceeds 50 g (confirmed after asking about the daily free sugar foods and drinks’ intake)? | Yes |
|  |  | No |
| 7 | Do you need assistance to perform your oral hygiene? | Yes |
|  |  | No |
| 8 | How often do you brush your teeth daily with fluoridated toothpaste? | < 2/day |
|  |  | ≥ 2/day |
| 9 | If you wear a denture, for how long have you wore it? | < 2 years |
|  |  | ≥ 2 years |
| 10 | If you wear a denture, how often do you clean it? | < 1/day |
|  |  | ≥ 1/day |
| 11 | If you wear a denture, what do you clean it with? | Soap |
|  |  | Dentifrice/mouthwash |
| 12 | If you wear a denture/s, do you remove it before bedtime? | Yes |
|  |  | No |
| 13 | Have you lately perceived the need for dental care? | Yes |
|  |  | No |
| 14 | What was the reason for your last dental visit? | Asymptomatic attendance |
|  |  | Symptomatic attendance |
| 15 | Do you currently feel painful sites in your mouth? | Yes |
|  |  | No |
| 16 | During the last three months, have you been concern about dental problems? | Yes |
|  |  | No |
| 17 | During the last three months, have you used medication due to dental pain or discomfort? | Yes |
|  |  | No |
| 18 | During the last three months, have you changed the type of food due to dental pain or discomfort? | Yes |
|  |  | No |

Additional file is a table listing the individual factors of the related questionnaire (based on validated tools) including 18 items: demographics (2 items), oral-health related practices/risk factors (10 items), oral-health related quality of life (5 items), and systemic condition (1 item).
